# Correction: Tonon et al. 5-Azacytidine Downregulates the Proliferation and Migration of Hepatocellular Carcinoma Cells In Vitro and In Vivo by Targeting miR-139-5p/ROCK2 Pathway. *Cancers* 2022, *14*, 1630

**DOI:** 10.3390/cancers18060922

**Published:** 2026-03-12

**Authors:** Federica Tonon, Maja Cemazar, Urska Kamensek, Cristina Zennaro, Gabriele Pozzato, Sergio Caserta, Flora Ascione, Mario Grassi, Stefano Guido, Cinzia Ferrari, Laura Cansolino, Francesco Trotta, Biljana Grcar Kuzmanov, Giancarlo Forte, Fabiana Martino, Francesca Perrone, Riccardo Bomben, Valter Gattei, Nicola Elvassore, Erminio Murano, Nhung Hai Truong, Michael Olson, Rossella Farra, Gabriele Grassi, Barbara Dapas

**Affiliations:** 1 Department of Life Sciences, Cattinara University Hospital, Trieste University, Strada di Fiume 447, I-34149 Trieste, Italy; ftonon@units.it (F.T.); fp385@cam.ac.uk (F.P.); bdapas@units.it (B.D.); 2 Department of Experimental Oncology, Institute of Oncology Ljubljana, Zaloska 2, SI-1000 Ljubljana, Slovenia; mcemazar@onko-i.si (M.C.); ukamensek@onko-i.si (U.K.); bkuzmanov@onko-i.si (B.G.K.); 3 Faculty of Health Sciences, University of Primorska, Polje 42, SI-6310 Izola, Slovenia; 4 Department of Medical, Surgical and Health Sciences, University of Trieste, Cattinara Hospital, Strada di Fiume 447, I-34149 Trieste, Italy; cristina.zennaro@asugi.sanita.fvg.it (C.Z.); gabriele.pozzato@asugi.sanita.fvg.it (G.P.); 5 Department of Chemical, Materials and Industrial Production Engineering, University of Naples “Federico II”, Piazzale V. Tecchio 80, I-80125 Naples, Italy; sergio.caserta@unina.it (S.C.); floriana.ascione@gmail.com (F.A.); stefano.guido@unina.it (S.G.); 6 CEINGE Advanced Biotechnologies, via Gaetano Salvatore, 486, I-80145 Napoli, Italy; 7 Department of Engineering and Architecture, University of Trieste, Via Valerio 6/A, I-34127 Trieste, Italy; mario.grassi@dia.units.it; 8 Department of Clinic-Surgical Sciences, Laboratory of Experimental Surgery and Animal Facility, University of Pavia, Via Ferrata 9, I-27100 Pavia, Italy; cinzia.ferrari@unipv.it (C.F.); lauracansolino@libero.it (L.C.); 9 Department of General Surgery, Maggiore Hospital, Largo Donatori del Sangue 1, I-26900 Lodi, Italy; ceccotrotta@libero.it; 10 International Clinical Research Center (ICRC) of St Anne’s University Hospital, CZ-65691 Brno, Czech Republic; giancarlo.forte@fnusa.cz (G.F.); fabianamartino@outlook.it (F.M.); 11 Department of Paediatrics, University of Cambridge, Addenbrooke’s Hospital, Hills Road, Cambridge CB2 0QQ, UK; 12 Clinical and Experimental Onco-Haematology Unit, Centro di Riferimento Oncologico, Istituto di Ricovero a Cura a Carattere Scientifico IRCCS, 33081 Aviano, Italy; rbomben@cro.it (R.B.); vgattei@cro.it (V.G.); 13 Industrial Engineering Department, University of Padova, Via Francesco Marzolo, 9, I-35131 Padova, Italy; nicola.elvassore@unipd.it; 14 Nealys SRL, Via Flavia 23/1, I-34148 Trieste, Italy; murano@nealys.com; 15 Stem Cell Research and Application Laboratory, VNUHCM, University of Science, Ho Chi Minh City 72711, Vietnam; thnhung@hcmus.edu.vn; 16 Department of Chemistry and Biology, X University, MaRS Discovery District, West Tower 661 University Avenue, Toronto, ON M5G 1M1, Canada

## Error in Figure

In the original publication [1], there was a mistake in Figure 2b as published. The wrong image was used for the Huh-7 control (2b tubulin). The corrected Figure 2b and appears below. The authors state that the scientific conclusions are unaffected. This correction was approved by the Academic Editor. The original publication has also been updated.

## Figures and Tables

**Figure 2:**
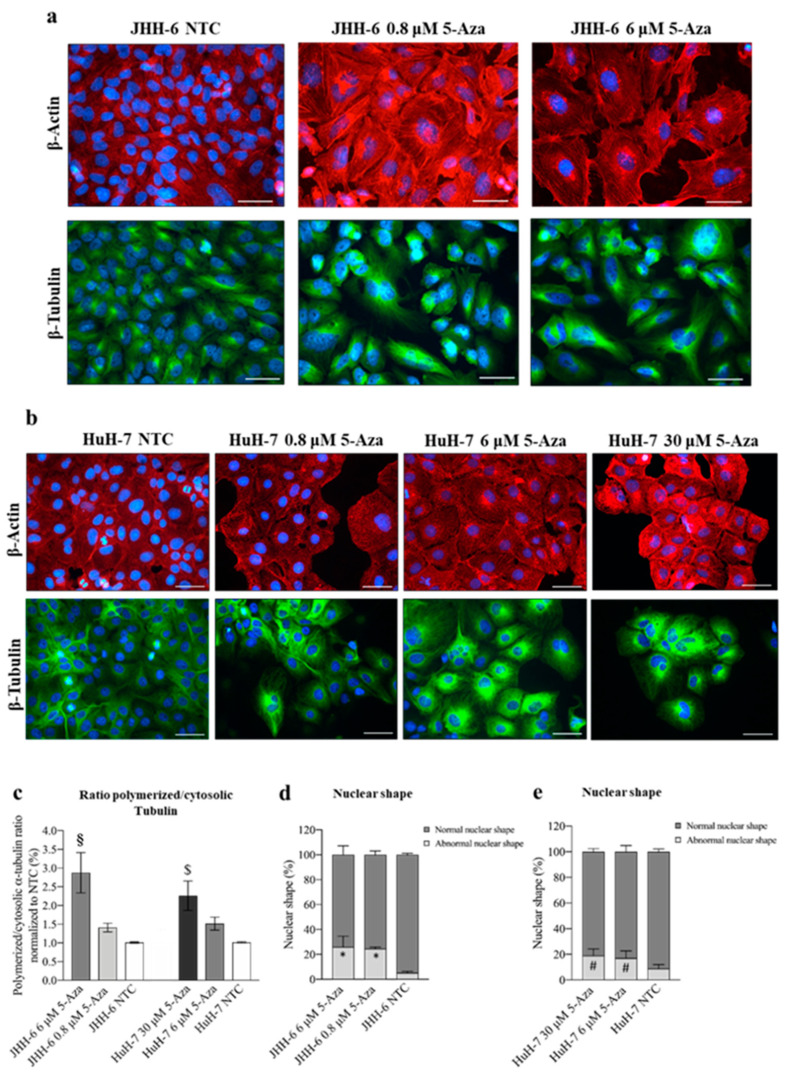
Effects of 5-Aza on cell cytoskeleton. (**a**,**b**) Immunostaining of β-actin and β-tubulin in JHH-6 and HuH-7 cells, respectively. Images were acquired with a Leica DM 2000 microscope. Red = β-actin; green = β-tubulin; blue = DAPI. Magnification 40X (bar = 100 μm). (**c**) Ratio of polymerized/cytosolic α-tubulin in 5-Aza treated vs. non-treated cells (NTC); data are expressed as mean ± SEM; JHH-6 NTC vs. JHH-6/5-Aza, ^§^ *p* = 0.04; HuH-7 NTC vs. HuH-7/5-Aza, ^$^ *p* = 0.045, *n* = 3. (**d**,**e**) Quantification of the number of altered nuclei shape in 5-Aza-treated vs. non-treated cells. Data, expressed as mean ± SEM, are reported as percentage normalized to the average of non-treated cells (NTC). Abnormal nuclei in JHH6/NTC vs. JHH6/0,8-6 μM, * *p* < 0.0001 *n* = 60; HuH7/NTC vs. HuH7/6-30 μM, ^#^ *p* < 0.001 *n* = 60.

## References

[1] Tonon F., Cemazar M., Kamensek U., Zennaro C., Pozzato G., Caserta S., Ascione F., Grassi M., Guido S., Ferrari C. (2022). 5-Azacytidine Downregulates the Proliferation and Migration of Hepatocellular Carcinoma Cells In Vitro and In Vivo by Targeting miR-139-5p/ROCK2 Pathway. Cancers.

