# Supplementary material for: 5-Azacytidine Downregulates the Proliferation and Migration of Hepatocellular Carcinoma Cells In Vitro and In Vivo by Targeting miR-139-5p/ROCK2 Pathway
Source: Cancers (Basel). 2022 Mar 23;14(7):1630. doi: 10.3390/cancers14071630 (PMC8996928; doi:10.3390/cancers14071630)

Figure 4

In red lanes reported
In the manuscript

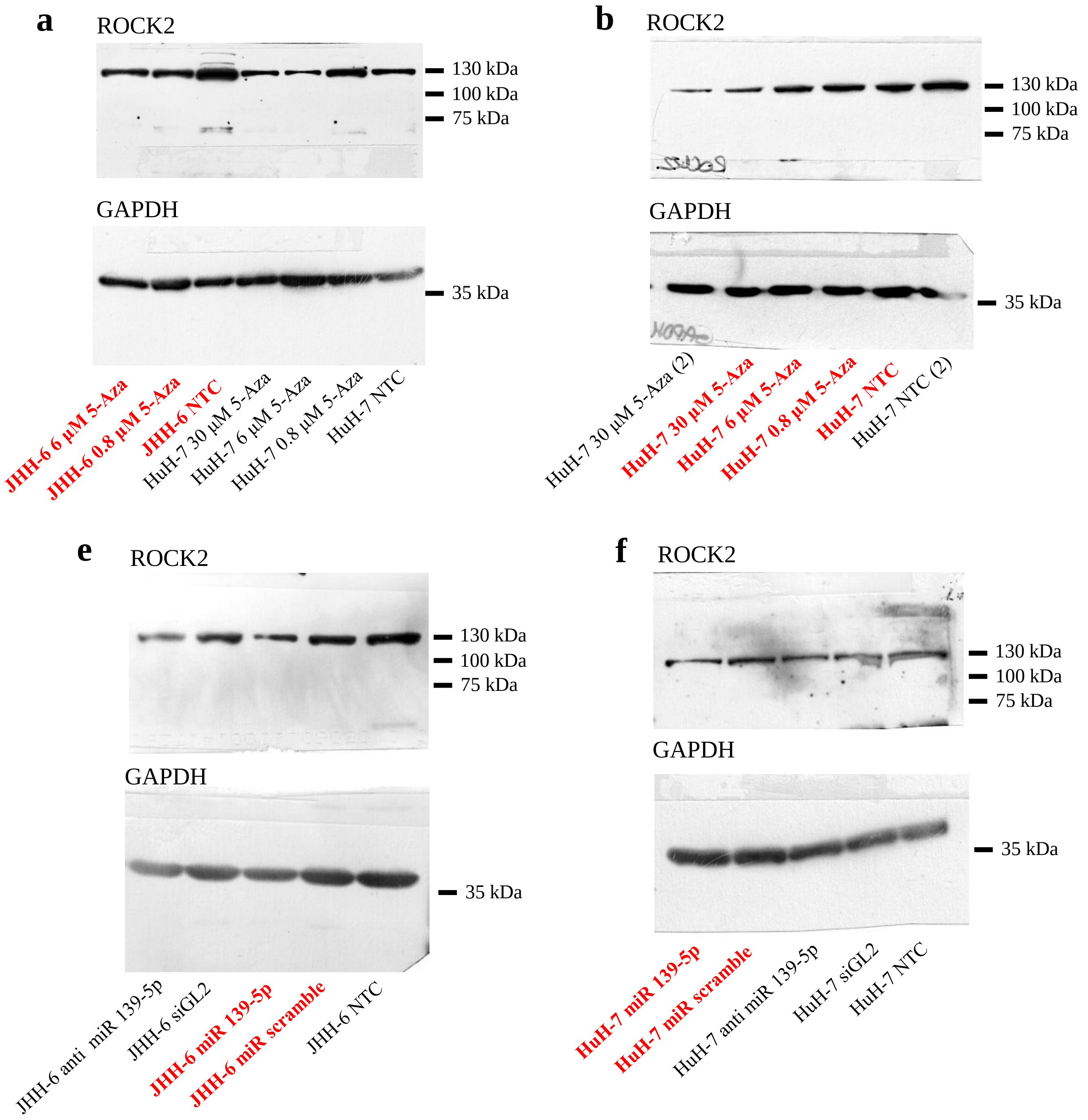

Figure 6

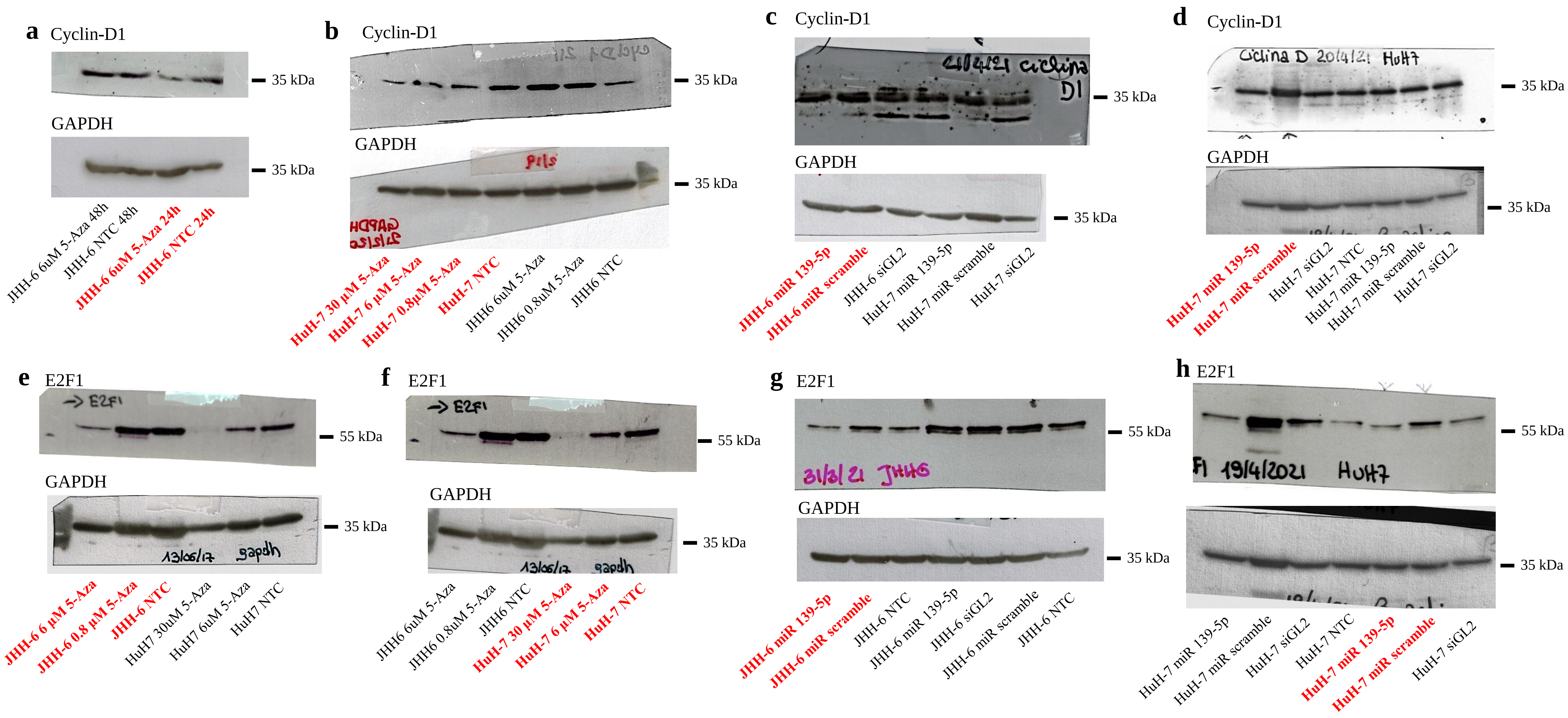
a
Cyclin-D1
35 kDa
GAPDH
35 kDa
JHH-6 6uM 5-Aza 48h
JHH-6 NTC 48h
JHH-6 6uM 5-Aza 24h
JHH-6 NTC 24h
b
Cyclin-D1
35 kDa
GAPDH
35 kDa
HuH-7 30 µM 5-Aza
HuH-7 6 µM 5-Aza
HuH-7 0.8µM 5-Aza
HuH-7 NTC
JHH6 6uM 5-Aza
JHH6 0.8uM 5-Aza
JHH6 NTC
c
Cyclin-D1
35 kDa
GAPDH
35 kDa
JHH-6 miR 139-5p
JHH-6 miR scramble
JHH-6 siGL2
HuH-7 miR 139-5p
HuH-7 miR scramble
HuH-7 siGL2
d
Cyclin-D1
35 kDa
GAPDH
35 kDa
HuH-7 miR 139-5p
HuH-7 miR scramble
HuH-7 siGL2
HuH-7 NTC
HuH-7 miR 139-5p
HuH-7 miR scramble
HuH-7 siGL2
e
E2F1
55 kDa
GAPDH
35 kDa
JHH-6 6 µM 5-Aza
JHH-6 0.8 µM 5-Aza
JHH-6 NTC
HuH7 30uM 5-Aza
HuH7 6uM 5-Aza
HuH7 NTC
f
E2F1
55 kDa
GAPDH
35 kDa
JHH6 6uM 5-Aza
JHH6 0.8uM 5-Aza
JHH6 NTC
HuH-7 30 µM 5-Aza
HuH-7 6 µM 5-Aza
HuH-7 NTC
g
E2F1
55 kDa
GAPDH
35 kDa
JHH-6 miR 139-5p
JHH-6 miR scramble
JHH-6 NTC
JHH-6 miR 139-5p
JHH-6 siGL2
JHH-6 miR scramble
JHH-6 NTC
h
E2F1
55 kDa
35 kDa
HuH-7 miR 139-5p
HuH-7 miR scramble
HuH-7 siGL2
HuH-7 NTC
HuH-7 miR 139-5p
HuH-7 miR scramble
HuH-7 siGL2

Figure 7

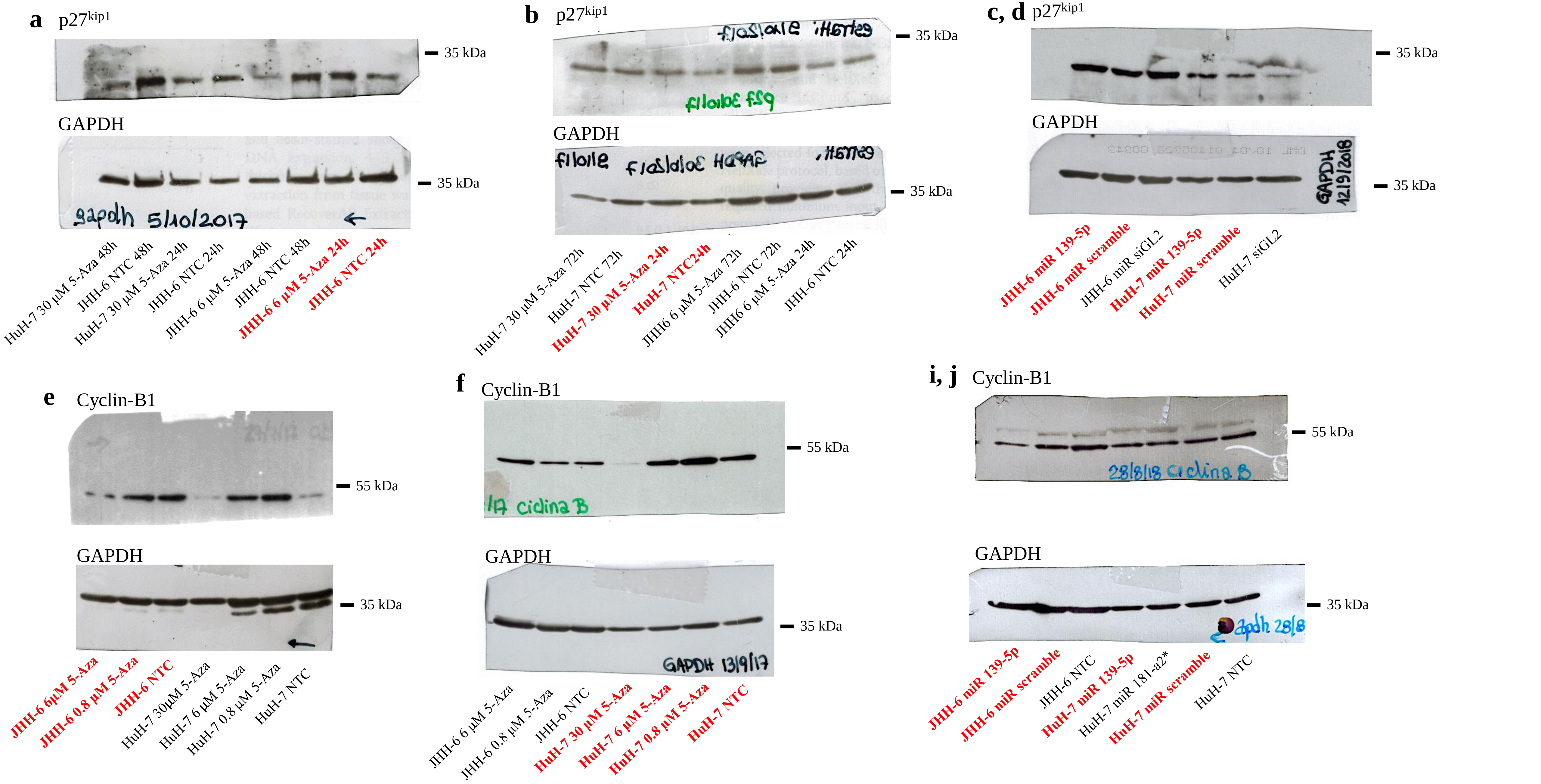
a
$p27^{kip1}$
35 kDa
GAPDH
35 kDa
HuH-7 30 µM 5-Aza 48h
JHH-6 NTC 48h
HuH-7 30 µM 5-Aza 24h
HuH-6 NTC 24h
JHH-6 6 µM 5-Aza 48h
JHH-6 NTC 48h
JHH-6 6 µM 5-Aza 24h
JHH-6 NTC 24h
b
$p27^{kip1}$
35 kDa
GAPDH
35 kDa
HuH-7 30 µM 5-Aza 72h
HuH-7 NTC 72h
HuH-7 30 µM 5-Aza 24h
HuH-7 NTC24h
JHH6 6 µM 5-Aza 72h
JHH-6 NTC 72h
JHH6 6 µM 5-Aza 24h
JHH-6 NTC 24h
c, d
$p27^{kip1}$
35 kDa
GAPDH
35 kDa
JHH-6 miR 139-5p
JHH-6 miR scramble
JHH-6 miR siGL2
HuH-7 miR 139-5p
HuH-7 miR scramble
HuH-7 siGL2
e
Cyclin-B1
55 kDa
GAPDH
35 kDa
JHH-6 6µM 5-Aza
JHH-6 0.8 µM 5-Aza
JHH-6 NTC
HuH-7 30µM 5-Aza
HuH-7 6 µM 5-Aza
HuH-7 0.8 µM 5-Aza
HuH-7 NTC
f
Cyclin-B1
55 kDa
GAPDH
35 kDa
JHH-6 6 µM 5-Aza
JHH-6 0.8 µM 5-Aza
JHH-6 NTC
HuH-7 30 µM 5-Aza
HuH-7 6 µM 5-Aza
HuH-7 0.8 µM 5-Aza
HuH-7 NTC
i, j
Cyclin-B1
55 kDa
GAPDH
35 kDa
JHH-6 miR 139-5p
JHH-6 miR scramble
JHH-6 NTC
HuH-7 miR 139-5p
HuH-7 miR 181-a2*
HuH-7 miR scramble
HuH-7 NTC

Figure 8

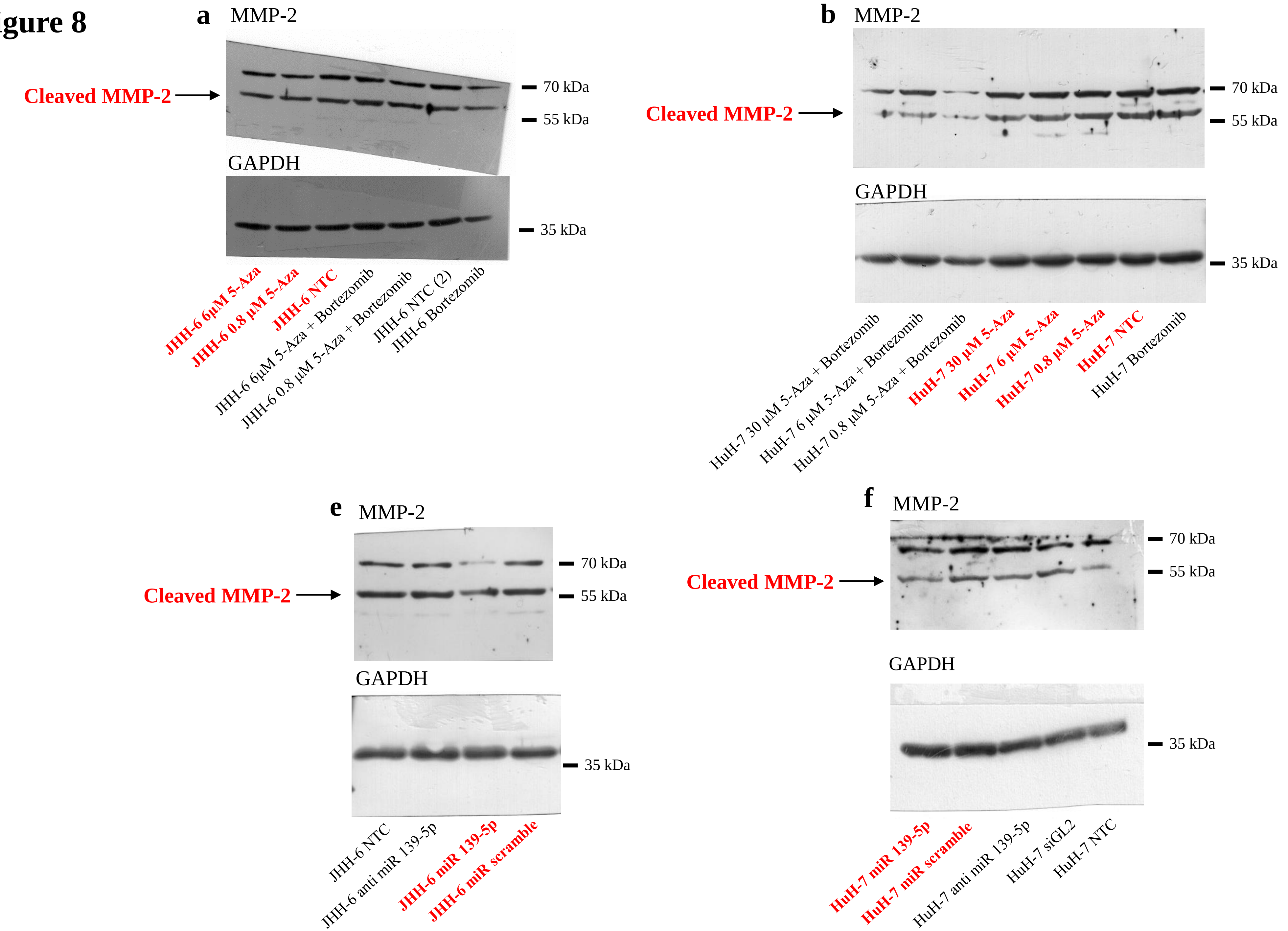
a
MMP-2
Cleaved MMP-2
70 kDa
55 kDa
GAPDH
35 kDa
JHH-6 6µM 5-Aza
JHH-6 0.8 µM 5-Aza
JHH-6 NTC
JHH-6 6µM 5-Aza + Bortezomib
JHH-6 0.8 µM 5-Aza + Bortezomib
JHH-6 NTC (2)
JHH-6 Bortezomib
b
MMP-2
Cleaved MMP-2
70 kDa
55 kDa
GAPDH
35 kDa
HuH-7 30 µM 5-Aza + Bortezomib
HuH-7 6 µM 5-Aza + Bortezomib
HuH-7 0.8 µM 5-Aza + Bortezomib
HuH-7 30 µM 5-Aza
HuH-7 6 µM 5-Aza
HuH-7 0.8 µM 5-Aza
HuH-7 NTC
HuH-7 Bortezomib
e
MMP-2
Cleaved MMP-2
70 kDa
55 kDa
GAPDH
35 kDa
JHH-6 NTC
JHH-6 anti miR 139-5p
JHH-6 miR 139-5p
JHH-6 miR scramble
f
MMP-2
Cleaved MMP-2
70 kDa
55 kDa
GAPDH
35 kDa
HuH-7 miR 139-5p
HuH-7 miR scramble
HuH-7 anti miR 139-5p
HuH-7 siGL2
HuH-7 NTC

Supplementary data 5

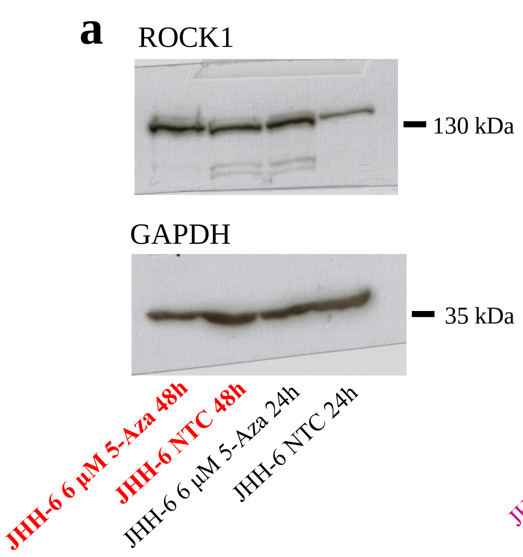

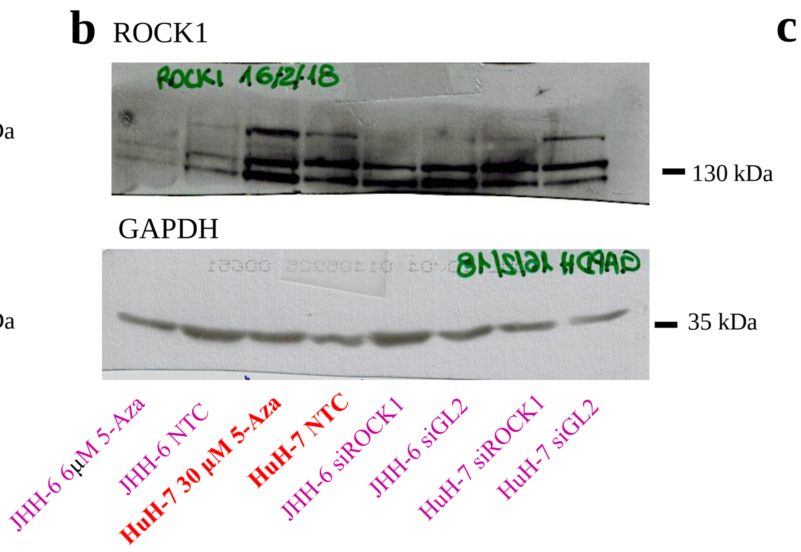

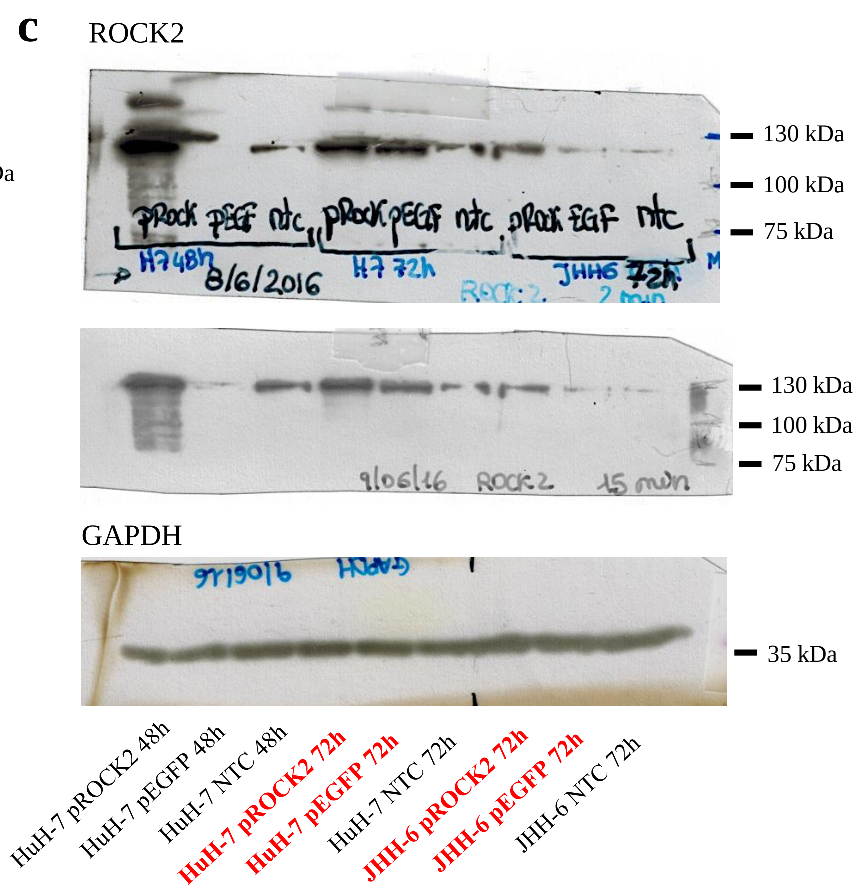

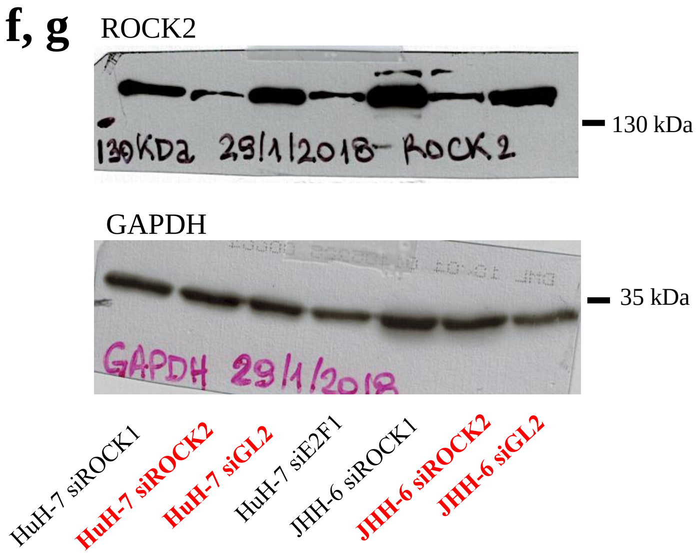

Supplement: Supplementary file 1 [file cancers-14-01630-s001.zip › cancers-1557545- original image.pdf]
