# Supplementary material for: 5-Azacytidine Downregulates the Proliferation and Migration of Hepatocellular Carcinoma Cells In Vitro and In Vivo by Targeting miR-139-5p/ROCK2 Pathway
Source: Cancers (Basel). 2022 Mar 23;14(7):1630. doi: 10.3390/cancers14071630 (PMC8996928; doi:10.3390/cancers14071630)
Supplement: Supplementary file 1 [file cancers-14-01630-s001.zip › cancers-1557545-supplementary- updated.pdf]

Figure S1

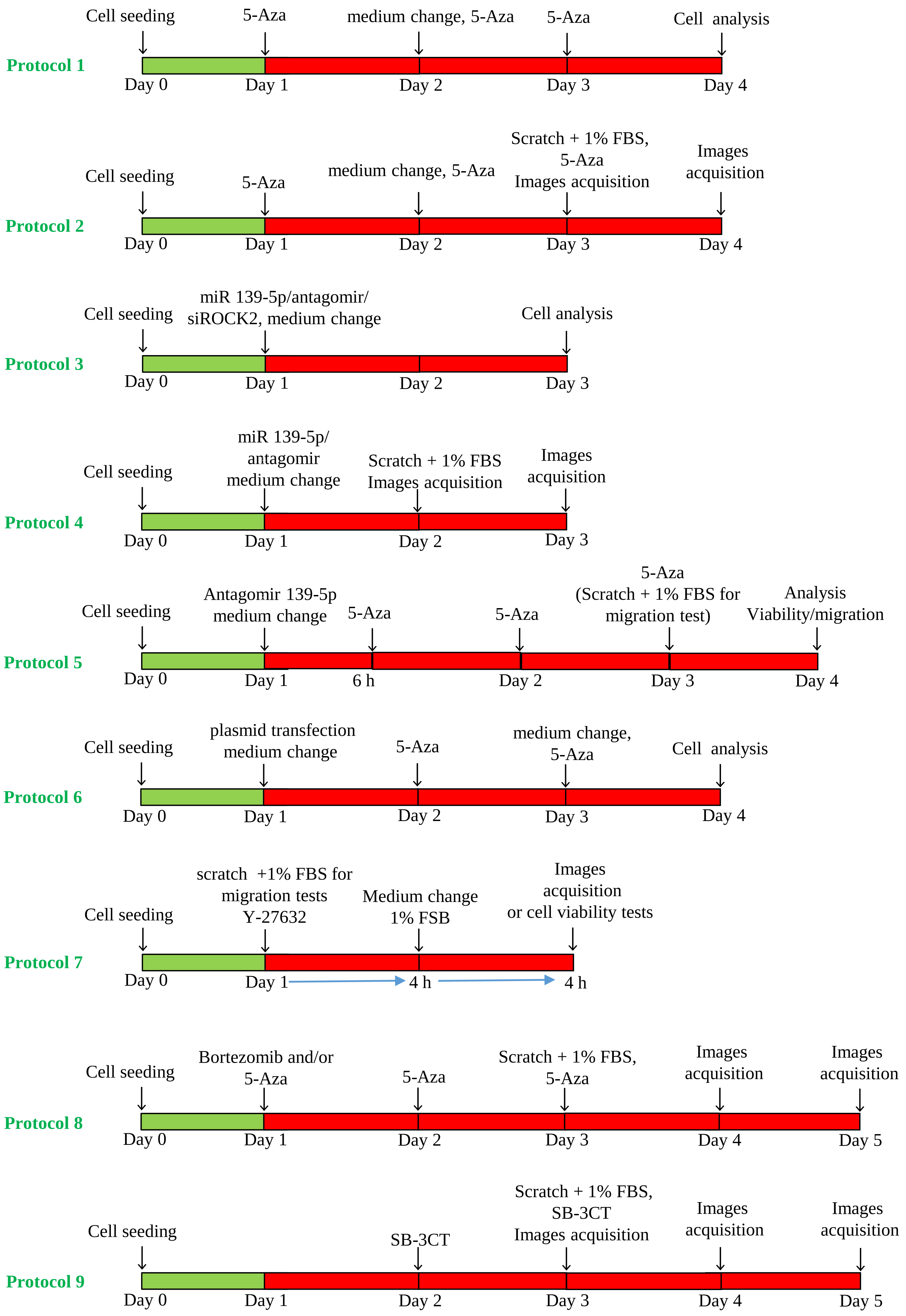
Protocol 1
Cell seeding
5-Aza
medium change, 5-Aza
5-Aza
Cell analysis
Day 0
Day 1
Day 2
Day 3
Day 4
Protocol 2
Cell seeding
5-Aza
medium change, 5-Aza
Scratch + 1% FBS,
5-Aza
Images acquisition
Images
acquisition
Day 0
Day 1
Day 2
Day 3
Day 4
Protocol 3
Cell seeding
miR 139-5p/antagomir/
siROCK2, medium change
Cell analysis
Day 0
Day 1
Day 2
Day 3
Protocol 4
Cell seeding
miR 139-5p/
antagomir
medium change
Scratch + 1% FBS
Images acquisition
Images
acquisition
Day 0
Day 1
Day 2
Day 3
Protocol 5
Cell seeding
Antagomir 139-5p
medium change
5-Aza
5-Aza
5-Aza
(Scratch + 1% FBS for
migration test)
Analysis
Viability/migration
Day 0
Day 1
6 h
Day 2
Day 3
Day 4
Protocol 6
Cell seeding
plasmid transfection
medium change
5-Aza
medium change,
5-Aza
Cell analysis
Day 0
Day 1
Day 2
Day 3
Day 4
Protocol 7
Cell seeding
scratch +1% FBS for
migration tests
Y-27632
Medium change
1% FSB
Images
acquisition
or cell viability tests
Day 0
Day 1
4 h
4 h
Protocol 8
Cell seeding
Bortezomib and/or
5-Aza
5-Aza
Scratch + 1% FBS,
5-Aza
Images
acquisition
Images
acquisition
Day 0
Day 1
Day 2
Day 3
Day 4
Day 5
Protocol 9
Cell seeding
SB-3CT
Scratch + 1% FBS,
SB-3CT
Images acquisition
Images
acquisition
Images
acquisition
Day 0
Day 1
Day 2
Day 3
Day 4
Day 5

Figure S2

Cell number

a

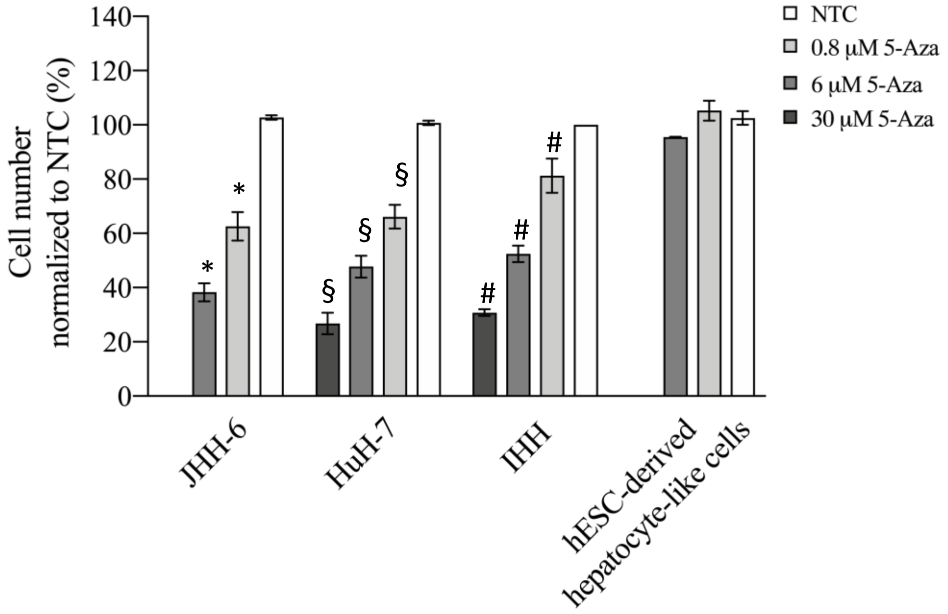

Cell viability

b

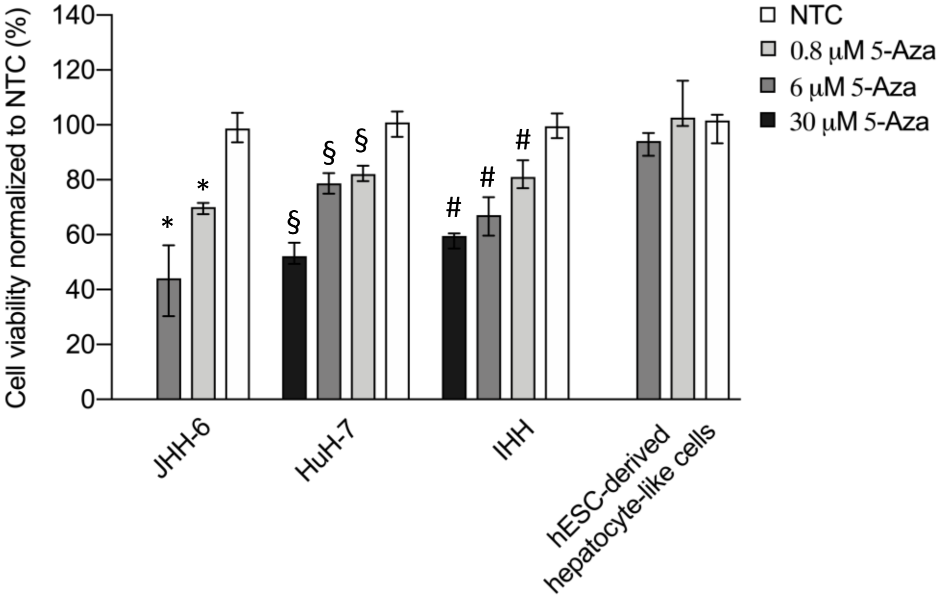

a) Effects on cell number. Data, expressed as mean ± SEM, are reported as percentage normalized to the average of NTC (Non Treated Cells). JHH-6/5-Aza vs JHH-6/NTC *$p<0.0001$, n=12; HuH-7/5-Aza vs HuH-7/NTC §$p< 0.0001$, n=10; IHH/5-Aza vs IHH/NTC, #$p< 0.0001$, n=10; hESC-derived hepatocyte-like cells/5-Aza vs hESC-derived hepatocyte-like cells/NTC, n=10. **b)** Effects on cell viability. Data are reported as percentage normalized to the average of NTC. JHH6/5-Aza vs JHH-6/NTC *$p<0.0001$, data are reported as mean ± SEM, n=10; HuH-7/5-Aza vs HuH-7/NTC §$p< 0.0001$, data expressed as mean ± SEM, n=6; IHH/5-Aza vs IHH/NTC, #$p< 0.0001$, n=10, data are reported as median with interquartile range; hESC-derived hepatocyte-like cells, n=3, data expressed as median with interquartile range.

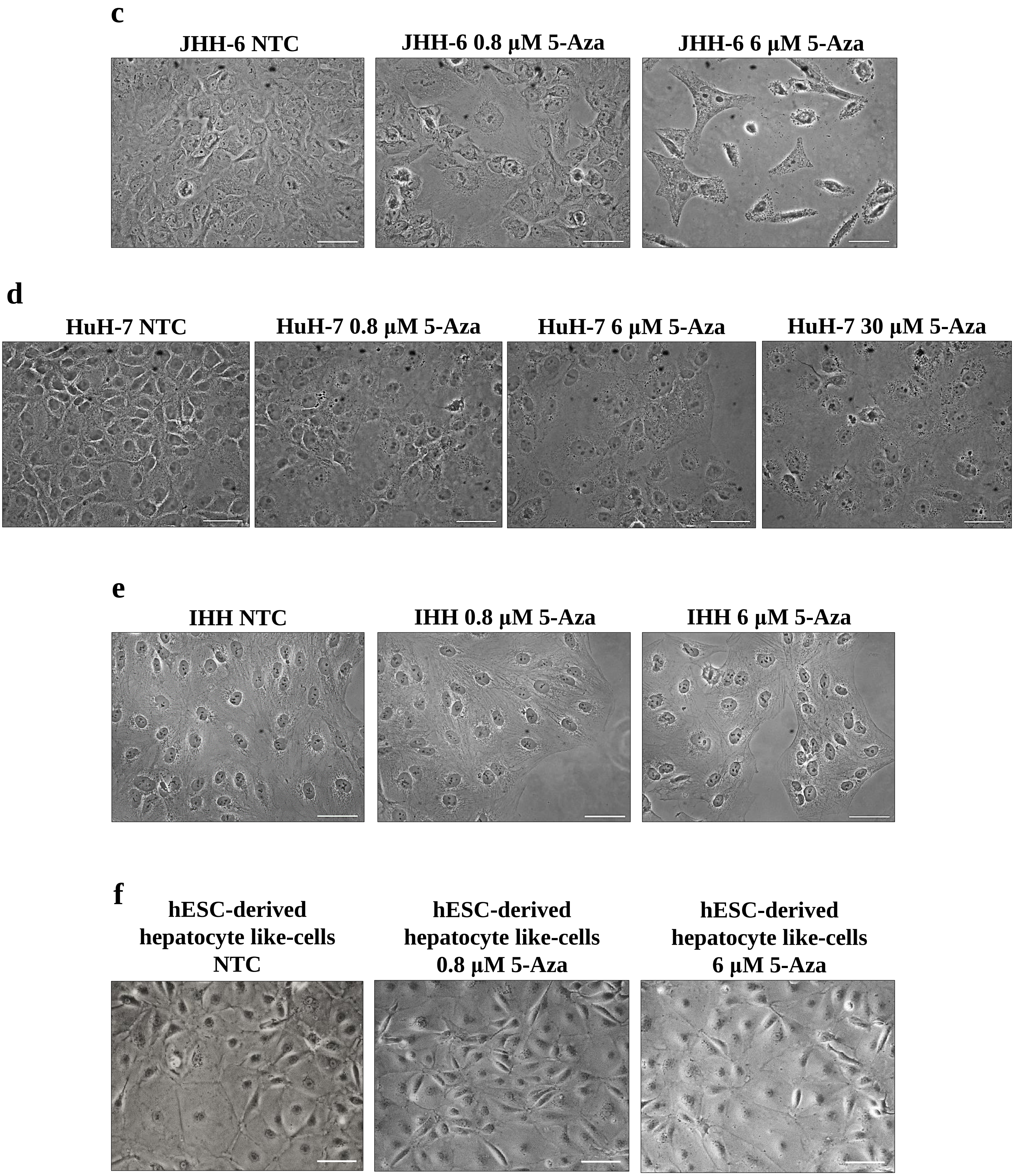

c-f) *Cell morphology following 5-Azacytidine treatment* **.** Images were acquired with phase-contrast microscope (Leica DM 2000). Magnification (bar = 50 µm).

Figure S3

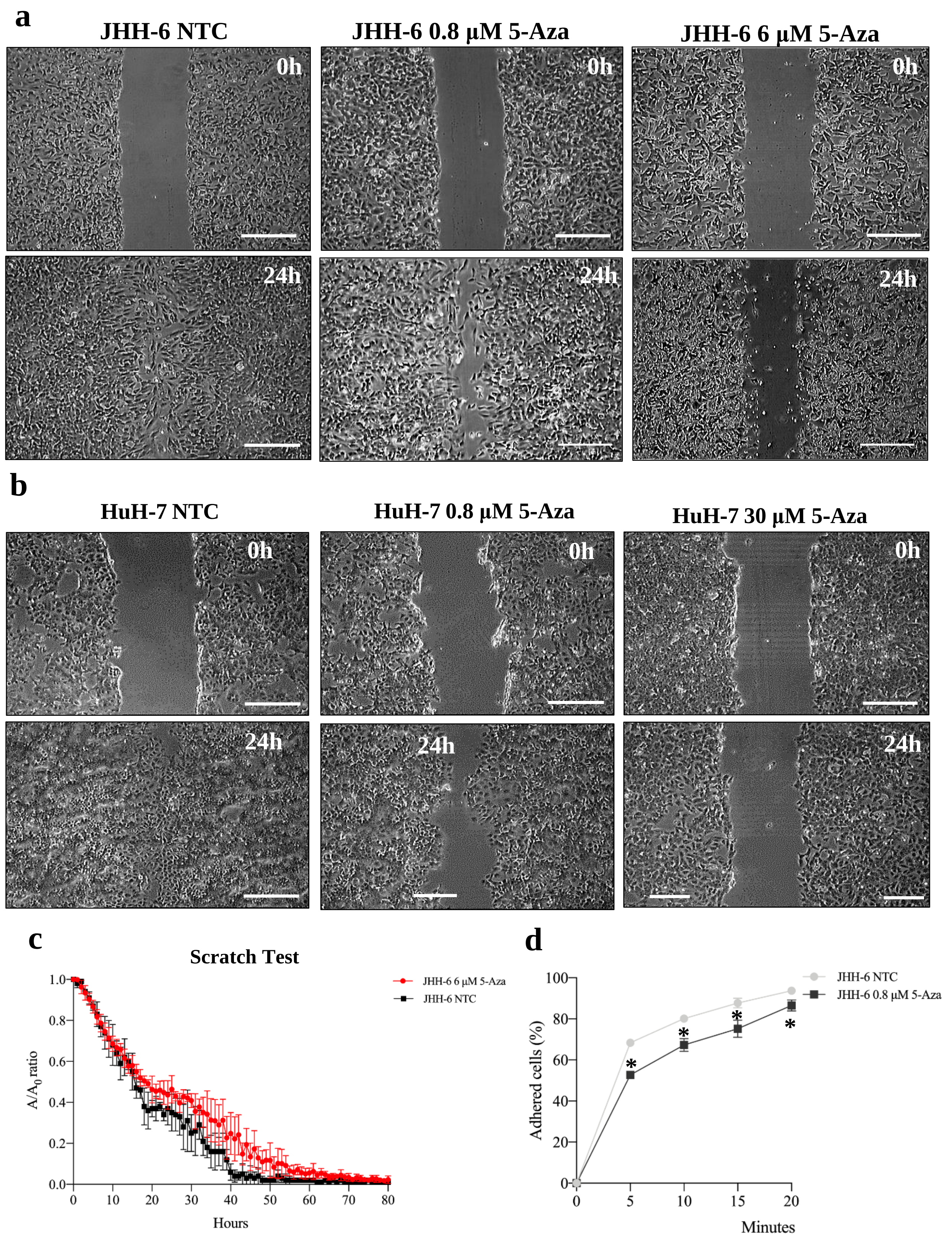

a-b) *JHH-6 and HuH-7 migration*. Representative images of scratch assay are reported. Images were acquired with Leica DM IRB microscope, (bar = 50µm). **C**) *Time-lapse analysis*. The kinetic of scratch closing was followed by time-laps in JHH-6, JHH-6 6µM 5-Aza vs JHH-6 NTC (non treated) p= 0.00001, data, expressed as A/A0, are represented as mean ± SD; A=area of the cell free region at a given time, A0 area at the beginning of the test). **D**) *JHH-6 adhesion*. Data are reported as mean ± SEM, JHH-6/5-Aza vs JHH-6/NTC *p<0.05, n=4.

Figure S4

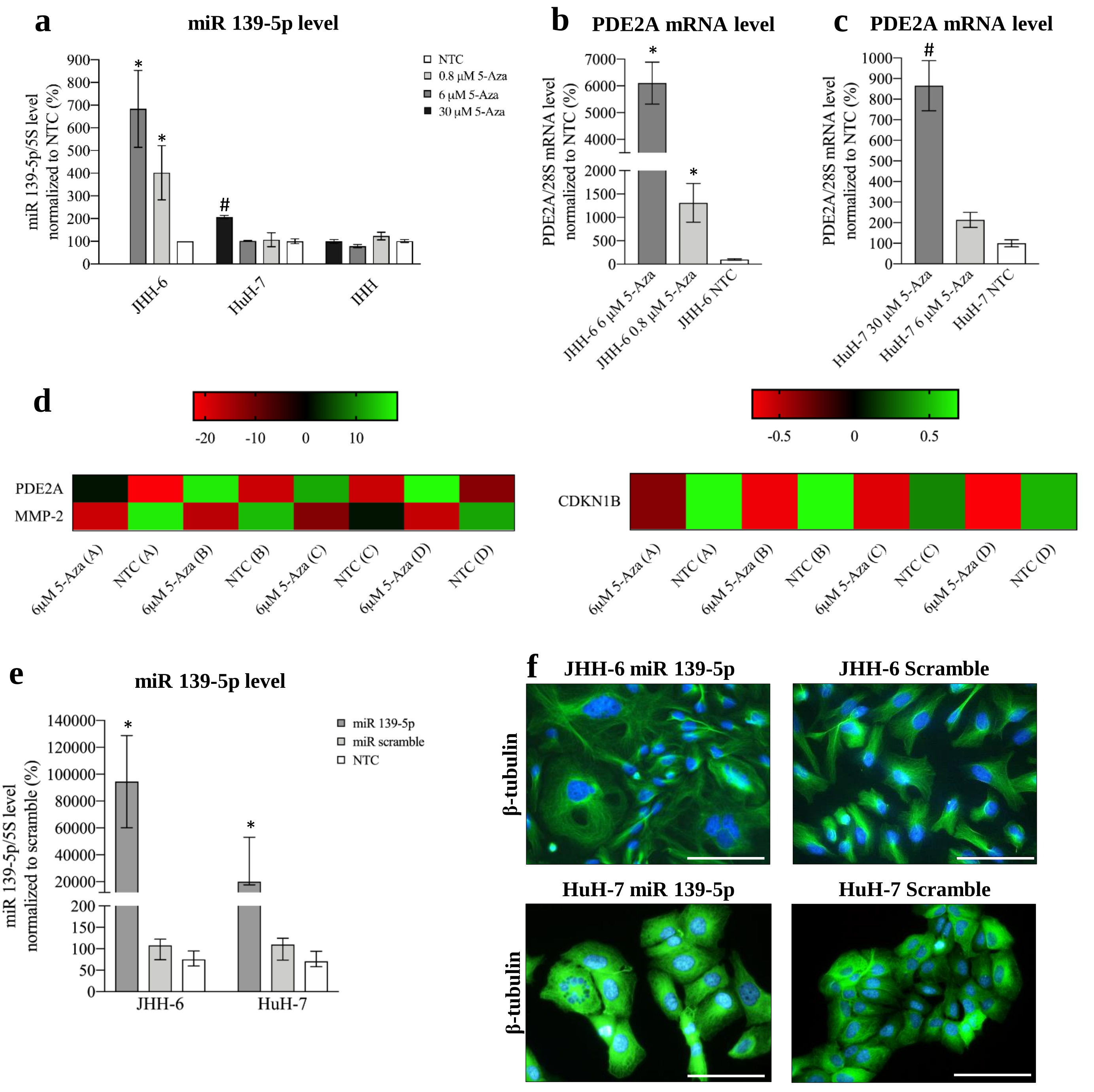

a) *miR 139-5p levels after 5-Aza treatment*. Data, expressed as percentage of NTC (non treated cells), are reported as mean ± SEM, n=9, NTC/JHH-6 vs 0,8-6µM/JHH-6 *p=0.0063; HuH-7/NTC vs 30 µM/JHH-6 #p=0.0001. **b,c**) *PDE2A levels after 5-Aza treatment*. Data, expressed as percentage of NTC, are represented as mean ± SEM, n=9, NTC/JHH-6 vs 0,8-6µM/JHH-6 *p<0.0015; NTC/HuH-7 vs 30 µM/HuH-7 #p=0.0034. **d)** *Expression levels of PDE2A, MMP-2 and CDKN1B (cyclin B1)*. Evaluation by microchip analysis in JHH-6 treated by 5-Aza (6µM) vs NTC; the data measured for each of the four couples of sample tested are reported (couples letter: A,B,C,D); the extent of the differential expression is quantified by a red-green scale above each measurement. **e)** *miR 139-5p levels following transfection*. Data are reported as median with interquartile range, scramble/JHH-6 vs miR 139-5p/JHH-6 and scramble/HuH-7 vs miR 139-5p/HuH-7, *p<0.0022, n=9. **f)** *Immunostaining of β-tubulin in JHH-6 (up) and HuH-7 (down) cells*. Images were acquired with Leica DM 2000 microscope. Green = β-tubulin, Blue=DAPI, bar=100µm.

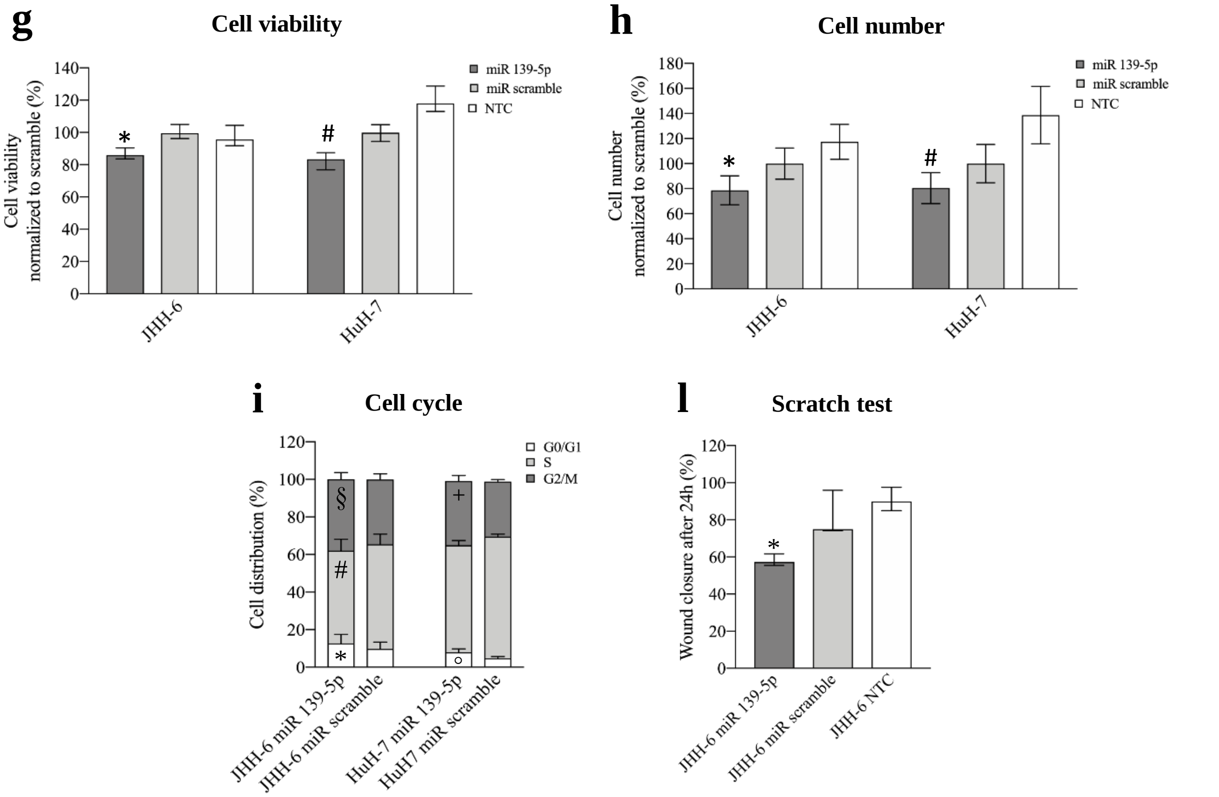

g*) JHH-6/HuH-7 cell viability (MTT assay) following miR 139-5p mimic transfection*. Data, normalized to the average of the miR scramble (scr), are represented as median with interquartile range, n=12, JHH-6/scr vs JHH-6/miR 139-5p *p=0.0001; HuH-7/scr vs HuH-7/miR 139-5p $^{\#}$p=0.0001. **h)** *JHH-6/HuH-7 cell number following miR 139-5p mimic transfection*. JHH-6/scr vs JHH-6/miR 139-5p *p=0.0078, n=8, data, normalized to the average of miR scr treated cells, are represented as mean ± SEM; HuH-7/scr vs HuH-7/miR 139-5p $^{\#}$p=0.0017, data, normalized to the average of scr treated cells, are reported as mean ± SEM. **i)** *Cell cycle phase distribution in JHH-6/HuH-7 after miR 139-5p mimic transfection*. JHH-6 scr G0/G1 vs JHH-6 miR 139-5p G0/G1 *p=0.0098, JHH-6 scr S vs JHH-6 miR 139-5p S $^{\#}$p=0.027, JHH-6 scr G2/M vs JHH-6 miR 139-5p G2/M §p=0.025, data, expressed as percentage, are represented as mean ± SEM, n=8; HuH-7 scr G0/G1 vs HuH-7 miR 139-5p G0/G1 ° p=0.0001, HuH-7 scr G2/M vs HuH-7 miR 139-5p G2/M $^{+}$p=0.049, data, expressed as percentage, are represented as mean ± SEM, n=6. **l)** *Migration of miR 139-5p mimic-transfected JHH-6 (scratch assay)*. Data, normalized to NTC, are represented as median with interquartile range, n=4, JHH-6 scr vs JHH-6 miR 139-5p *p=0.0001.

Figure S5

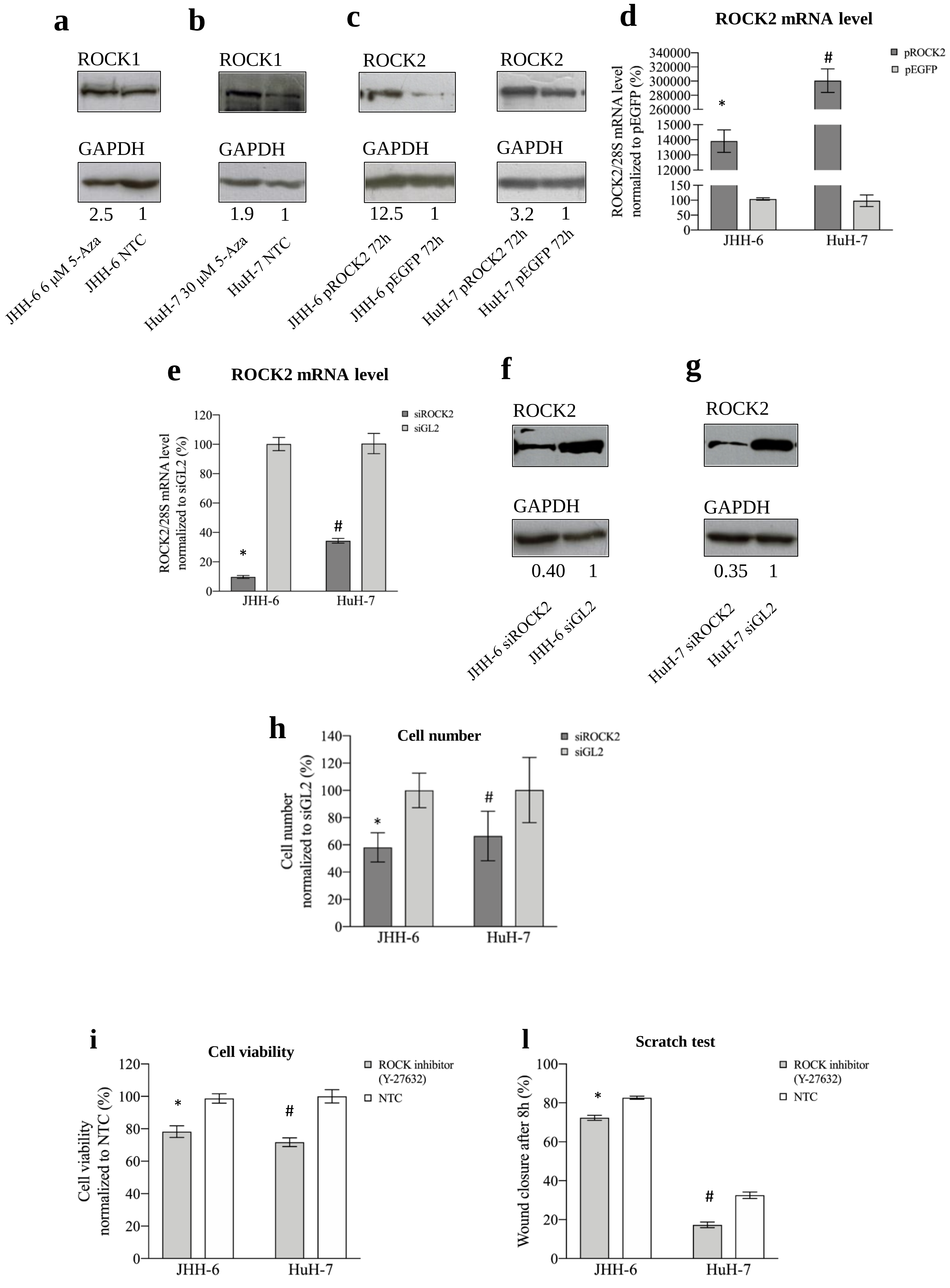

a
ROCK1
GAPDH
2.5 1
JHH-6 6 µM 5-Aza
JHH-6 NTC
b
ROCK1
GAPDH
1.9 1
HuH-7 30 µM 5-Aza
HuH-7 NTC
c
ROCK2
GAPDH
12.5 1
JHH-6 pROCK2 72h
JHH-6 pEGFP 72h
ROCK2
GAPDH
3.2 1
HuH-7 pROCK2 72h
HuH-7 pEGFP 72h
d
ROCK2 mRNA level
ROCK2/28S mRNA level normalized to pEGFP (%)
pROCK2
pEGFP
JHH-6
HuH-7
e
ROCK2 mRNA level
ROCK2/28S mRNA level normalized to siGL2 (%)
siROCK2
siGL2
JHH-6
HuH-7
f
ROCK2
GAPDH
0.40 1
JHH-6 siROCK2
JHH-6 siGL2
g
ROCK2
GAPDH
0.35 1
HuH-7 siROCK2
HuH-7 siGL2
h
Cell number
Cell number normalized to siGL2 (%)
siROCK2
siGL2
JHH-6
HuH-7
i
Cell viability
Cell viability normalized to NTC (%)
ROCK inhibitor (Y-27632)
NTC
JHH-6
HuH-7
l
Scratch test
Wound closure after 8h (%)
ROCK inhibitor (Y-27632)
NTC
JHH-6
HuH-7

m

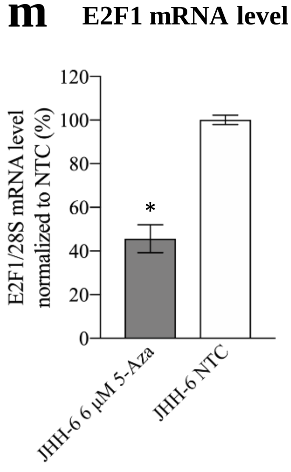

n

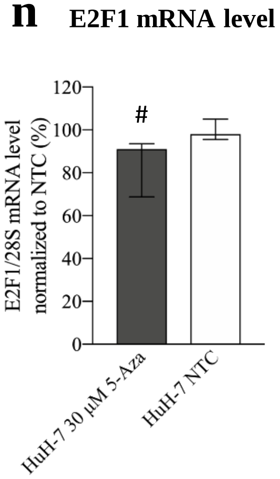

a,**b)** *Effects of 5-Aza on ROCK1 protein level*. Representative blots: below each blot, the ratio ROCK1/GAPDH, normalized to non treated cells (NTC), is reported. **c**) *ROCK2 protein level following ROCK2 overexpression*. Representative blots: the ratio ROCK2/GAPDH, normalized to cells over-expressing EGFP (pEGFP, control), is reported (72 h after transfection). **d**) *ROCK2 mRNA level following ROCK2 overexpression.* Data normalized to 28S levels and to the average of pEGFP treated cells (control), are reported in % as mean ± SEM, n=9; JHH-6 transfected by pEGFP vs JHH-6 transfected by pROCK2 *p=0.0001; HuH7 transfected by pEGFP vs transfected by pROCK2 # p=0.0001; pEGFP/pROCK2 = plasmids expressing EGFP/ROCK2 respectively. **e)** *ROCK2 silencing by siRNA (siROCK2)*. Data normalized to 28S levels and to the average of siGL2 (control siRNA), are reported in % as mean ± SEM, n=3, JHH-6/siGL2 vs JHH-6/siROCK2 *p=0.0001; HuH-7/siGL2 vs HuH-7/siROCK2 #p=0.0001. **f,g**) *Effects of siROCK2 on ROCK2 protein*. Representative blots: below each blot, the ratio ROCK2/GAPDH, normalized to siGL2 treated cells (control), is reported. **h)** *Cell number following ROCK2 down regulation by siROCK2*. Left: ROCK2 silencing in JHH-6; data in %, normalized to the average of siGL2 (control siRNA) treated JHH-6 are expressed as mean ± SEM, n=4, siROCK2 vs siGL2 *p=0.021. Right: ROCK2 silencing in HuH-7; data in %, normalized to the average of siGL2 treated HuH-7, are expressed as mean ± SEM, n=3, siROCK2 vs siGL2 $^{\#}$p=0.006. **i)** *Effects of ROCK2 inhibition by Y-27632 on cell viabi*lity. Left: data in %, normalized to the average of non-treated cells (NTC) are expressed as mean ± SEM, n=12, Y-27632/JHH-6 vs NTC/JHH-6 *p=0.0002. Right: data in %, normalized to the average of NTC are expressed as mean ± SEM, n=7, Y-27632/HuH-7 vs NTC/HuH-7 $^{\#}$p=0.0002. **l)** *Effects of ROCK2 inhibition by Y-27632 on cell migration (scratch assay)*. Left: data in %, normalized to the average of NTC are expressed as mean ± SEM, n=3, Y-27632 JHH-6 vs NTC JHH-6 *p=0.0001. Right: data in %, normalized to the average of NTC are expressed as mean ± SEM, n=3, Y-27632/HuH-7 vs NTC/HuH-7 $^{\#}$p=0.0001. **m,n**) *Effects of 5-Aza on E2F1 mRNA level*. Data normalized to 28S levels and to the average of NTC, are reported as mean ± SEM (JHH-6) or as median with interquartile range (HuH-7), n=6, NTC/JHH-6 vs 6 μM/JHH6 *p=0.0001, NTC/HuH-7 vs 30 μM/HuH-7 #p=0.0065.

Figure S6

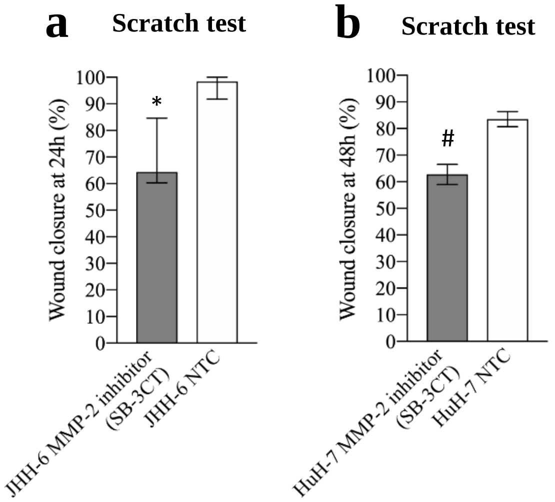

a,b) *Scratch assay following MMP-2 inhibitor SB-3CT administration.* Data, expressed as percentage normalized to the average of NTC (Non Treated Cells), are reported as median with interquartile range (JHH-6) or as median ± SEM (HuH-7); NTC/JHH-6 vs SB-3CT/JHH-6 *p=0.0001, NTC/HuH-7 vs SB-3CT/HuH-7 #p=0.002, n=3.

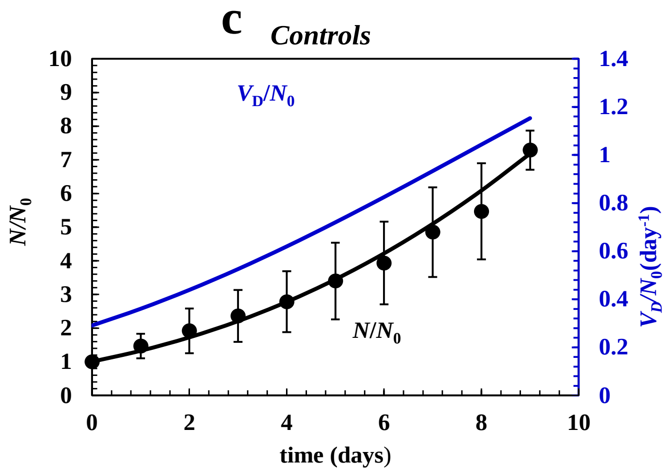

c) Best fitting of eq.(3) (black line) to tumor volume increase (black dots) referring to control samples depicted in Figure 9a. N/N_0 is the ratio between cells number N (proportional to tumor volume) at time t and at the beginning (N_0, day 0). V_D/N_0 represents the number of cells generated from each original cell after a given time. Vertical bars indicate data standard deviation. Fitting parameters read: k_D = (0.290 ± 0.023) day^{-1} and k_D = (0.066 ± 0.021) day^{-1}. Best fitting reliability is ensured by the F_{test}: F(1,8,095) < 956. Blue line represents the velocity of tumor volume increase evaluated according to eq.(4) and expressing the number of cells generated by each original cell after time t.

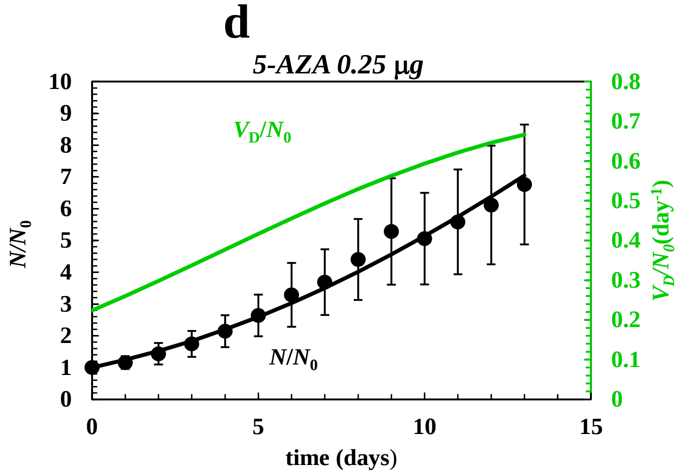

d) Best fitting of eq.(3) (black line) to tumor volume data (black dots) referring to animals treated with 5-AZA 0.25 µg (see Figure 9a). N/N_0 is the ratio between cells number N (proportional to tumor volume) at time t and at the beginning (N_0, day 0). V_D/N_0 represents the number of cells generated from each original cell after a given time. Vertical bars indicate data standard deviation. Fitting parameters read: k_D = (0.224 ± 0.012) day^{-1} and k_f = (0.066 ± 0.013) day^{-1}. Best fitting reliability is ensured by the F_{test}: F(1,12,095) < 716. Green line represents the velocity of tumor volume increase evaluated according to eq.(4) and expressing the number of cells generated by each original cell after time t.

e

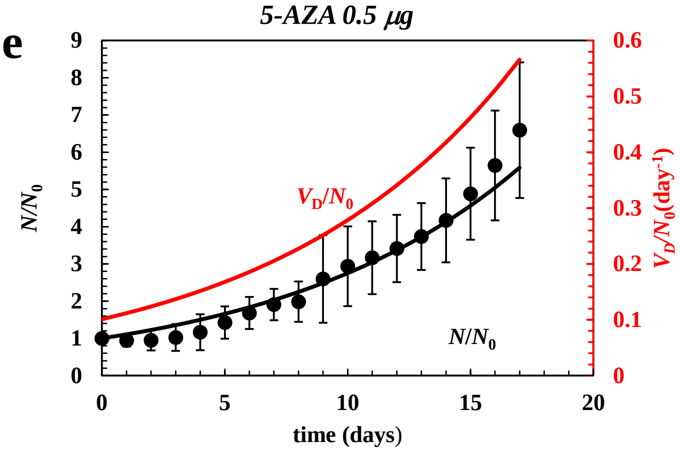

e) Best fitting of eq.(3) (black line) to tumor volume data (black dots) referring to animals treated with 5-Aza 0.5 μg (see Figure 9a). N/N_0 is the ratio between cells number N (proportional to tumor volume) at time t and at the beginning (N_0, day 0). V_D/N_0 represents the number of cells generated from each original cell after a given time. Vertical bars indicate data standard deviation. Fitting parameters read: k_D = (0.063 ± 0.005) day^{-1} and k_f = 0 day^{-1}. Best fitting reliability is ensured by the F_{test}: $F(1,16,095) < 24$. Red line represents the velocity of tumor volume increase evaluated according to eq.(4) and expressing the number of cells generated by each original cell after time t.

f

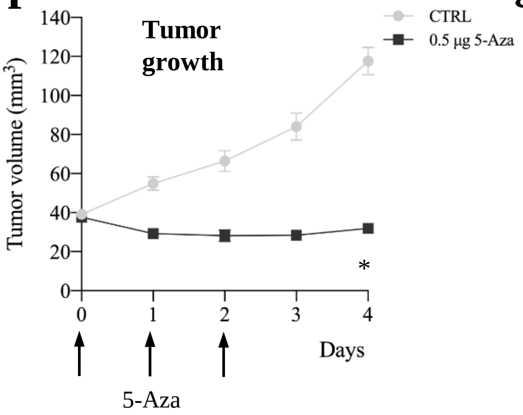

g

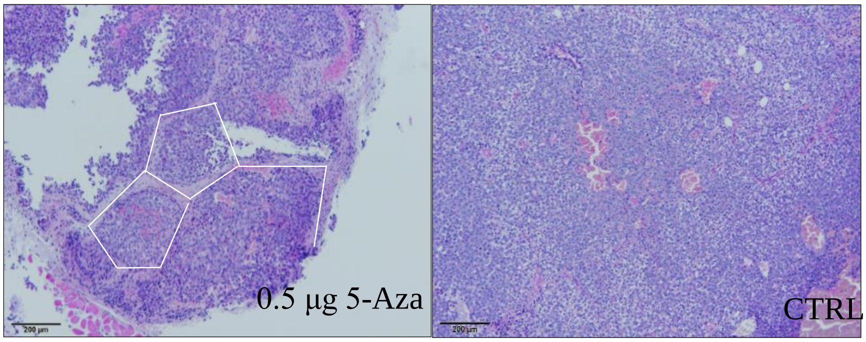

h

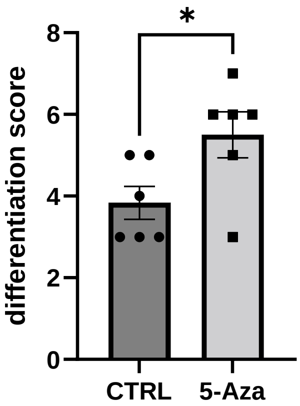

i

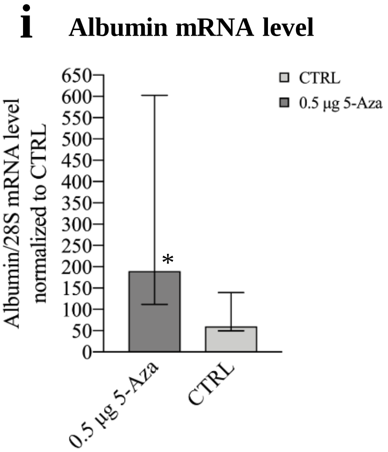

f) *Effects of 5-Aza on tumor growth.* Comparison of tumor growth trend between 5-Aza-treated and control mice (CRTL). The arrows indicate the administration times for 5-Aza (0,5 μg per injection); data are reported in mm3 as median ± SEM , n=5, 5-Aza/day1-4 vs CRTL day/1-4 p*=0.044. **g)** Representative images of hematoxylin-eosin stained tumor tissues from the short-term experiment (day 4). Left, tumor injected by 5-Aza, the white lines indicate the border of hepatic-like psedo-lobules; right control non injected tumor; scale bar=200μm. **h**) *Effect of 5-Aza on tumor differentiation (day 4)*. A differentiation score was a sum of changes evaluating the differentation of tumor cells, presence of fibrotic tissue and formation of pseudolobules. Data are reported as mean ± SEM, n=6, CRTL vs 5-Aza *p=0.0366. **i**) *Effect of 5-Aza on albumin RNA level (day 4)*. Data normalized to 28S levels and to the average of CTRL, are reported in % as median with interquartile range, n=18, CRTL vs 5-Aza *p=0.0016.

Figure S7

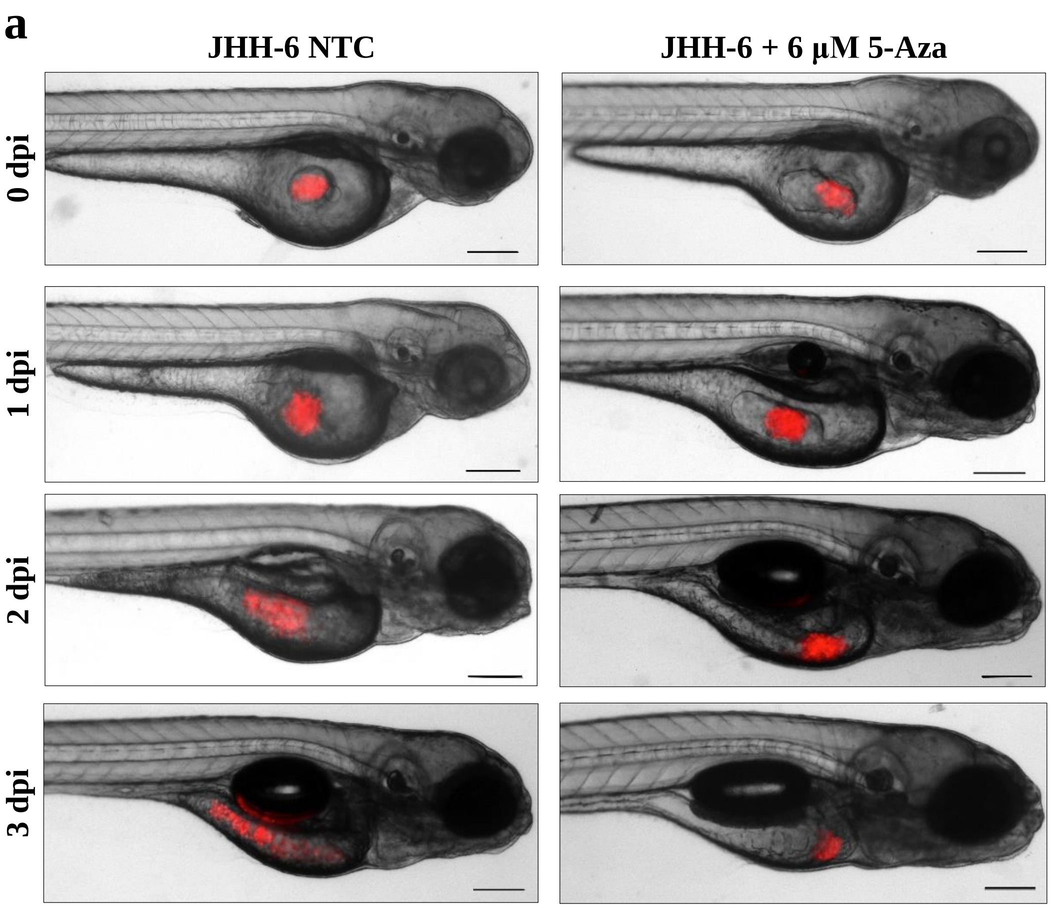

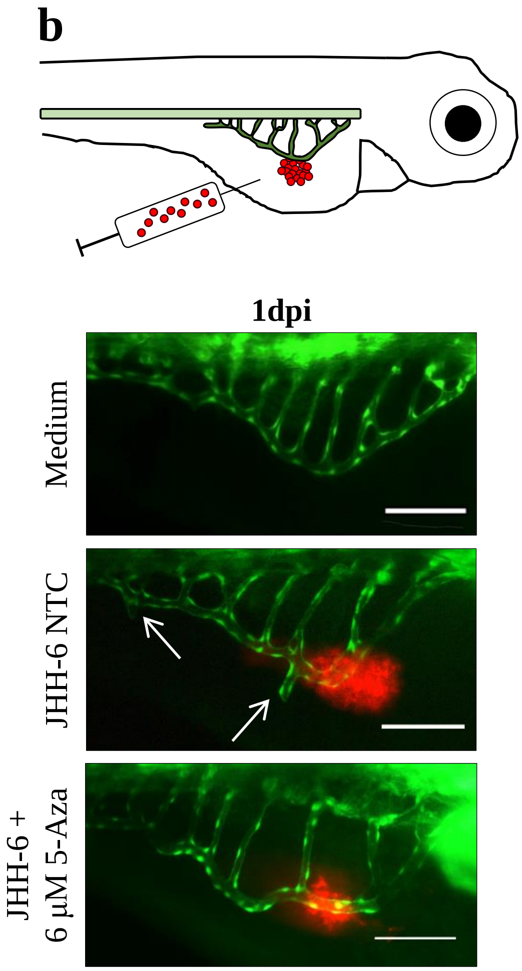

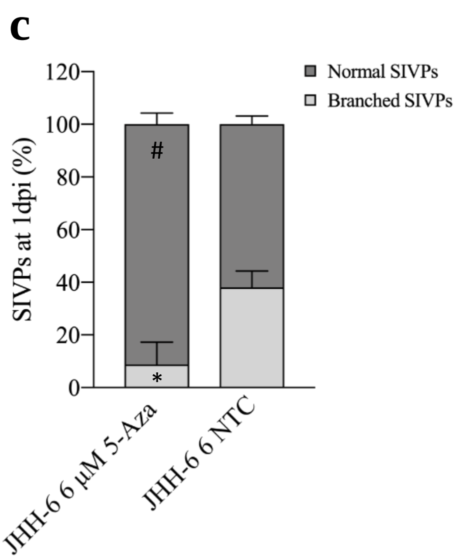

a) Representative visible light imagines of JHH-6 (stained in red) injected into embryos treated or non-treated by 5-Aza; dpi= days post cell injection, bar = 100 µm. **b)** Up: schematic representation of the JHH-6 (red) injection in the yolk of the Tg(fli1:EGFP) zebrafish strain that expresses in endothelial cells the enhanced green fluorescence protein; down: representative fluorescence imagines of JHH-6 (red) injected in animals treated or non-treated by 5-Aza; as control, animals injected with medium only are reported; the white arrows show new branches of SIVP (SubIntestinal Vessels Plexus), bar = 100 µm. **c)** Quantification of SIVP branching in treated (5-Aza) and non-treated animals, data are shown as mean ± SEM, n=45, branched SIVP/NTC-JHH-6 vs branched SIVP/5Aza-JHH-6 *p=0.0122, normal SIVP/NTC-JHH-6 vs normal SIVP/5-Aza-JHH-6 #p=0.0122,

Figure S8

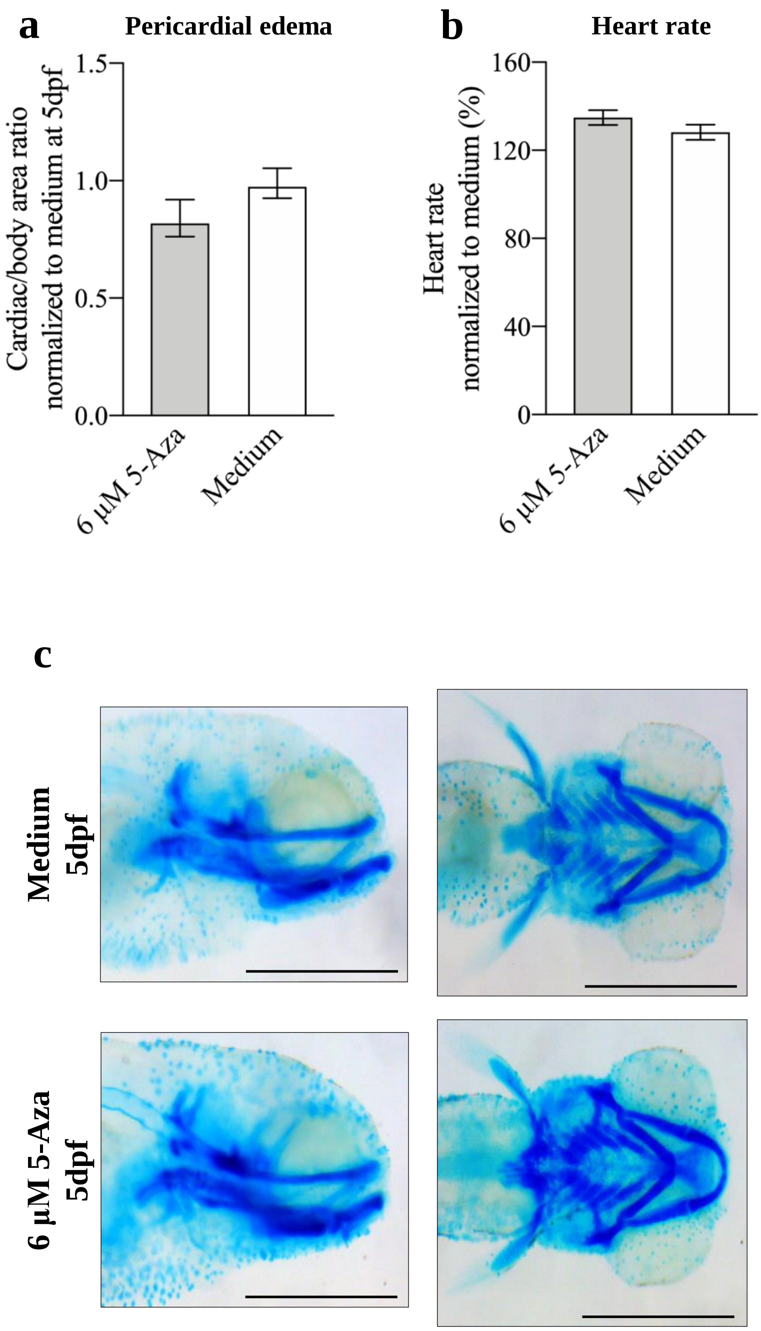

a,b) *Evaluation of possible 5-Aza cardiac toxic effects in zebrafish*. In A the effects on pericardial edema were evaluated; data, are expressed as median with interquartile range, n=30. In B the effects on heart rate were evaluated; data, are expressed as median with interquartile range, n=30. **c)** *Evaluation of possible 5-Aza toxic effects on the osteo-cartilaginous system*. Reported are representative imagines of the osteo-cartilagineous system (labelled by alcian blue solution) in non-treated (up) and 5-Aza treated animals (down), dpi= days post cell injection, bar = 500 µm.

Figure S9

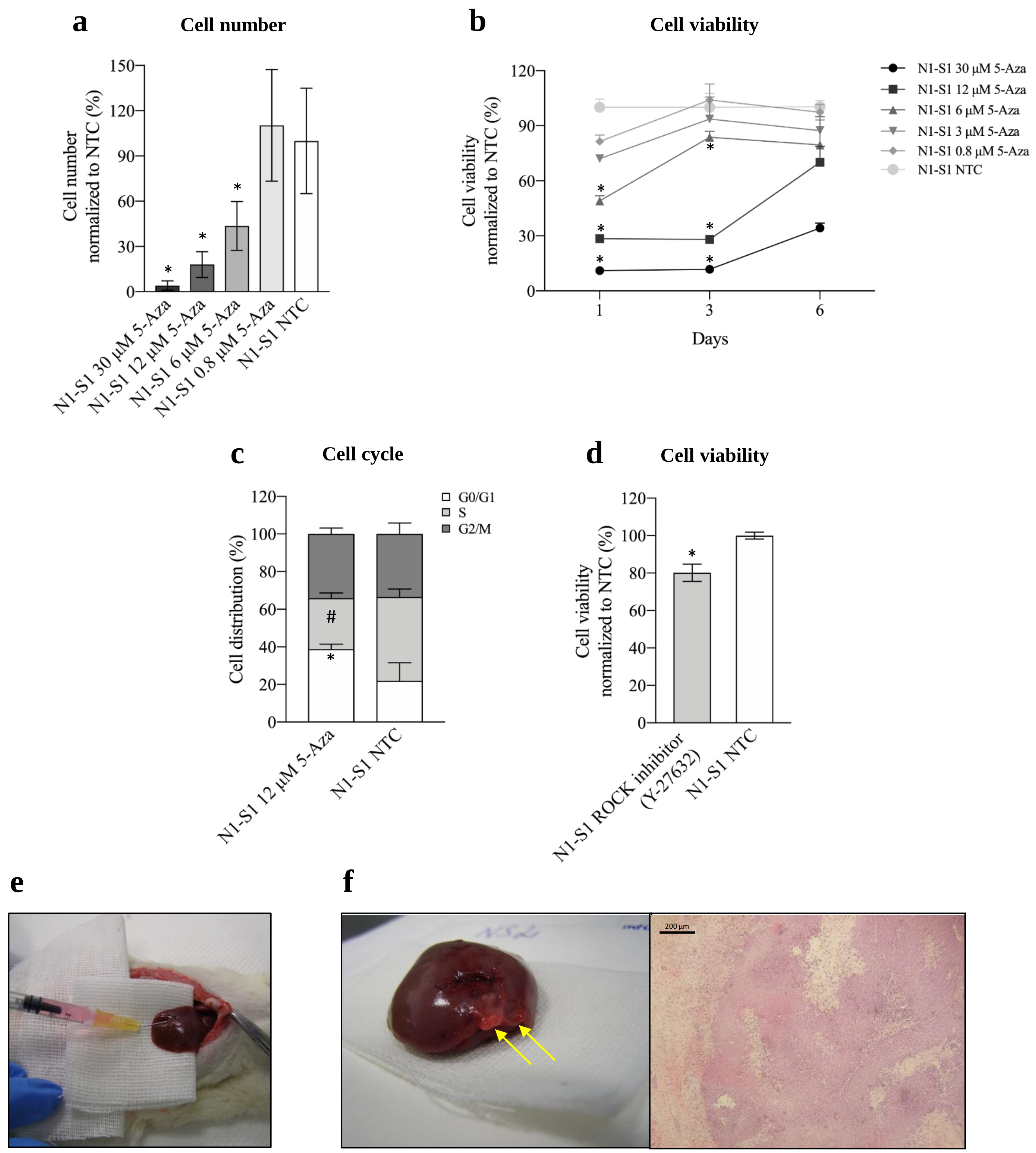

a) *Effects of 5-Aza on N1-S1 number*. Data, expressed as percentage normalized to the average of NTC (Non Treated Cells), are reported as mean ± SEM, n=6, N1-S1 treated by 6/12/30 μM 5-Aza vs N1S1 NTC *p<0.0034. ***b)*** *Effects of 5-Aza on N1-S1 viability*. Data, expressed as percentage normalized to the average of NTC are reported as mean ± SEM, n=9, N1-S1 treated by 6/12/30 μM 5-Aza vs N1S1 NTC *p<0.006. ***c)*** *Effects of 5-Aza on N1-S1 cell cycle phase distribution*. Data, expressed as percentage, are reported as mean ± SEM, n=5, G0/G1-NTC vs G0/G1-12 μM * p=0.034, S-NTC vs S-12 μM # p=0.018. ***d)*** *Effect of ROCK2 inhibitor Y-27632 on N1-S1 viability*. Data, expressed as percentage normalized to the average of NTC, are reported as mean ± SEM, n=7, Y-27632/N1S1 vs NTC/N1-S1 * p=0.0052. **e)** *Inoculum of N1-S1 in the rat liver*. ***f)*** *Detection of tumor nodules in rat liver*. A representative imagine of macroscopic (left, arrows) and histology (hematoxylin-eosin stained, scale bar=250 μm) features of tumor nodules.
